# Relationship between red blood cell distribution width-to-albumin ratio and outcome of septic patients with atrial fibrillation: a retrospective cohort study

**DOI:** 10.1186/s12872-022-02975-1

**Published:** 2022-12-09

**Authors:** You-lan Gu, Duo Yang, Zhi-bin Huang, Yan Chen, Zai-shen Dai

**Affiliations:** 1 Department of Anesthesiology, Guangzhou Panyu Maternal Child Health Hospital (Guangzhou Panyu District He Xian Memorial Hospital), No. 2 Qinghe East Road, Shiqiao Street, Guangzhou, 511400 China; 2 Department of Anesthesiology, Jieyang People’s Hospital, NO.107 Tianfu Road, Rongcheng street, 522000 Jieyang, China; 3 Department of Anesthesiology, Maoming People’s Hospital, No.101 Weimin Road, Maonan street, 525000 Maoming, China

**Keywords:** Red blood cell distribution width, Albumin, Atrial fibrillation, Sepsis, Retrospective cohort study, MIMIC-IV

## Abstract

**Background:**

This retrospective cohort study aimed to investigate the association between red blood cell distribution width-to-albumin ratio (RAR) and in-hospital mortality in patients with sepsis and atrial fibrillation (AF).

**Methods:**

Data were obtained from the Medical Information Mart for the Intensive Care Database IV database version 1.0. Multivariate Cox regression models, curve-fitting, and Kaplan**–**Meier analyses were performed to determine the correlation between RAR and in-hospital mortality in patients with sepsis and AF.

**Results:**

This study included 3042 patients with sepsis and AF. Confounding variables were adjusted for in the Multivariable Cox regression analysis models. RAR was independently associated with in-hospital mortality (hazard ratio 1.06; 95% confidence interval 1.03–1.08; *p* < 0.001). A linear relationship was found between the RAR and in-hospital mortality in patients with sepsis and AF.

**Conclusion:**

Elevated RAR levels are associated with increased in-hospital mortality in patients with sepsis and AF. Further research is required to confirm this association.

**Supplementary Information:**

The online version contains supplementary material available at 10.1186/s12872-022-02975-1.

## Background

Atrial fibrillation (AF) is a common complication in intensive care units (ICU) with an incidence of 30% [1, 2]. Furthermore, patients with sepsis are particularly vulnerable to developing AF, with the incidence of new-onset AF ranging from 23 to 40%, as per recent data [3]. Notably, patients with sepsis and AF exhibit a greater risk of in-hospital and ICU mortality than patients with sepsis without AF [4, 5]. Thus, early identification of these patients could potentially result in better management, earlier targeted therapy, and higher survival rates.

The red cell distribution width (RDW) is a measure of the variability in the size of red blood cells (RBCs), and it increases during systemic inflammation [4]. RDW has been linked to clinical outcomes in a variety of clinical settings [5–7]. Some studies have reported RDW to be associated with outcomes in critically ill patients, including those with sepsis and septic shock [8, 9]. Wan et al. [10] reported that RDW levels influenced all-cause mortality and a composite of major adverse events in patients with AF. Serum albumin concentration reflects the host nutritional and inflammatory status [11] and is associated with the prognosis of patients with sepsis [12].

The RBC distribution width-to-albumin ratio (RAR, %/g/dL) is a novel inflammatory biomarker, which is equal to the RDW divided by the serum albumin level. Previous studies have also indicated that RAR is associated with mortality in patients with heart failure [13], aortic aneurysms [14], stroke [15], acute respiratory distress syndrome (ARDS) [16], diabetic ketoacidosis [17], and cancer [18]. Moreover, RAR is thought to accurately reflect inflammation and could be a key metric for evaluating criticality scores for cardiovascular disease in intensive care patients. However, the association between RAR in patients with sepsis and AF remains unclear.

The goal of this study was to investigate the relationship between the RAR and in-hospital mortality in patients with sepsis and AF.

## Methods

### Data source

This retrospective cohort study used data from the Medical Information Mart for Intensive Care (MIMIC)-IV database (version 1.0) [19]. MIMIC-IV, an update of MIMIC-III, includes data on 76,540 ICU stays between 2008 and 2019. Youlan Gu obtained approval to use this database (certification number 48844482). The data were anonymized, and the institutional review boards of the Massachusetts Institute of Technology (No. 0403000206) and Beth Israel Deaconess Medical Center (2001-P-001699/14) approved the use of the database for research.

### Study participants selection criteria

**Fig. 1:**
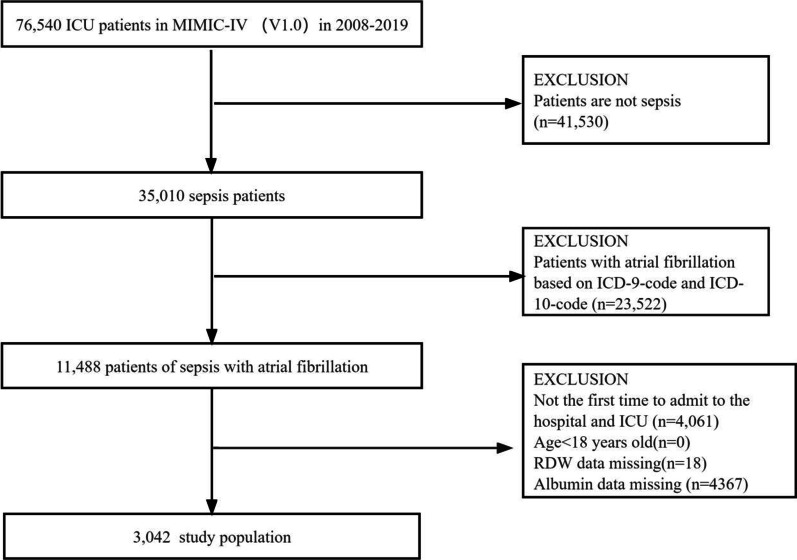
Flowchart of study patients. * ICU* intensive care database, *MIMIC* medical information mart for intensive care, *RAR* red blood cell distribution width-to albumin ratio

All adult patients (age > 18 years) with sepsis and AF were enrolled in this study. The diagnostic criteria for sepsis were consistent with sepsis 3.0, which is defined when the following conditions are met: documented or suspected infection and an acute increase in sequential organ failure assessment (SOFA) score of ≥ 2. Suspected infections were identified as prescriptions for antibiotics and sampling of bodily fluids for microbiological culture [20]. AF was defined as per ICD-9 codes 42,731 or ICD-10 codes I48 (Fig. 1). We adopted the date of the first ICU admission only for patients admitted to the ICU more than once. Conversely, patients who lacked data of interest, such as albumin and RDW values during hospitalization, were excluded from the study.

### Variable extraction

The study variables included demographic characteristics (age and sex), vital signs, laboratory parameters, organ support therapy, and comorbidities. The vital signs included heart rate, systolic blood pressure (SBP), diastolic blood pressure (DBP), mean arterial pressure (MAP), body temperature, and oxygen saturation (SPO_2_). Laboratory parameters included white blood cell (WBC) count, hemoglobin, hematocrit, platelet count, anion gap, serum bicarbonate, serum creatinine, blood urea nitrogen (BUN), glucose level, chloride, sodium, international normalized ratio (INR), plasma prothrombin time (PT), potassium, RDW, and serum albumin level within 24 h of ICU admission. Comorbidities included myocardial infarction (MI), congestive heart failure (CHF), peripheral vascular disease, cerebrovascular disease, dementia, chronic pulmonary disease, rheumatic disease, peptic ulcer disease, chronic liver disease, diabetes, paraplegia, renal disease, malignant cancer, metastatic solid tumor, acquired immune deficiency syndrome, mechanical ventilation (MV), and renal replacement therapy (RRT). Organ support therapy included MV and RRT, and illness severity was measured using the simplified Oxford Acute Severity of Illness Score (OASIS), Simplified Acute Physiology Score (SAPS II), and SOFA score. The survival information and length of stay data were gathered from a table titled “demographic ICU stay detail” in the MIMIC-IV database.

### Outcome

The primary outcome was in-hospital mortality, which was defined as survival status at hospital discharge. Patients without outcome information were excluded from the final cohort.

### Sensitivity analysis

Patients with hepatorenal syndrome prior to ICU admission were excluded as they may regularly receive large amounts of albumin during intravenous infusion [21].

### Statistical analysis

Patient characteristics were analyzed according to RAR quartiles. Data were expressed as mean ± standard deviation (SD) or median (quartile 1–quartile 3 [i.e., interquartile range (IQR)]) for continuous variables and as frequency or percentage for categorical variables. We used the chi-square test, one-way ANOVA, and the Kruskal**–**Wallis test to compare categorical, normally distributed, and nonnormally distributed continuous variables, respectively.

Univariate and multivariate Cox regression models were used to estimate the association between the RAR and in-hospital mortality in patients with sepsis and AF. Three models were used: model 1, adjusted for age and sex; model 2, adjusted for sex, age, MI, CHF, cerebrovascular disease, chronic pulmonary disease, rheumatic disease, diabetes, renal disease, chronic liver disease, MV, and RRT classification; and model 3, based on model 1 and model 2, but further adjusted for serum hematocrit, MBP, temperature, SPO_2_, potassium, INR, PT, and SOFA score.

Hospital survival was assessed using Kaplan–Meier survival curves according to RAR quartiles and evaluated using the log-rank test.

Furthermore, curve fitting was used to assess the linear relationship between RAR and in-hospital mortality in patients with AF and sepsis. To identify modifications and interactions, a stratified linear regression model and likelihood ratio test were used in the subgroup analyses. All analyses were performed using R 3.3.2 (http://www.R-project.org, The R Foundation) and Free Statistics version (version 1.7). Differences were considered statistically significant at a two-sided *p* < 0.05.

## Results

### Patient baseline characteristics

A total of 3042 eligible patients were identified based on the predetermined inclusion criteria (Fig. 1). The baseline characteristics of the patients are summarized in Table 1. The enrolled patients were grouped by RAR quartiles as follows: Q1, < 4.06; Q2, ≥ 4.06 and < 4.89; Q3, ≥ 4.89 and < 6; Q4, ≥ 6. The patients were aged 74.9 ± 12.3 years and included 1260 (41.4%) women and 1782 (58.6%) men. At the end of the median follow-up of 12.52 days, 789 (25.94%) patients died. Patients with a high RAR level tended to have a higher heart rate, RDW, WBC count, INR, and PT. They also had lower blood pressure, albumin, hemoglobin, hematocrit, platelet count, and serum glucose. Patients in the high RAR group were more likely to have CHF, cerebrovascular disease, chronic pulmonary disease, peptic ulcer disease, chronic liver disease, malignant cancer, metastatic solid tumors, and RRT. In addition, these patients had significantly higher OASIS, SAPSII, and SOFA scores (Table 1).

**Table 1:** Characteristics of the study patients

Characteristics	RAR				*P* value
	Q1 (< 4.06)	Q2 (4.06–4.89)	Q3 (4.89-6.0)	Q4 (≥ 6.0)	
	N = 761	N = 760	N = 760	N = 761	
*Demographics*					
Age, years	75.1 ± 12.2	76.0 ± 12.2	75.3 ± 12.1	73.3 ± 12.7	< 0.001
*Sex, n*					0.072
Male	476 (62.5)	440 (57.9)	437 (57.5)	429 (56.4)	
Female	285 (37.5)	320 (42.1)	323 (42.5)	332 (43.6)	
Heart rate (beats/min)	89.2 ± 22.0	92.1 ± 21.9	94.0 ± 22.5	97.2 ± 24.5	< 0.001
SBP (mmHg)	128.5 ± 24.8	122.7 ± 24.4	118.7 ± 24.2	113.5 ± 24.1	< 0.001
DBP (mmHg)	72.1 ± 19.3	67.9 ± 19.3	66.6 ± 19.0	64.0 ± 18.0	< 0.001
MBP (mmHg)	88.2 ± 21.2	82.4 ± 19.3	80.3 ± 19.2	77.0 ± 18.5	< 0.001
Temperature (℃)	36.7 ± 0.8	36.7 ± 0.9	36.7 ± 0.9	36.6 ± 1.1	0.151
SPO_2_ (%)	96.0 ± 4.8	96.2 ± 4.7	96.0 ± 4.6	95.7 ± 5.9	0.242
*Laboratory parameters*					
WBC count (10^9^/L)	11.4 (8.1, 16.0)	11.8 (8.3, 16.5)	12.5 (8.5, 18.0)	12.9 (7.7, 19.4)	0.005
Hemoglobin (g/dl)	12.7 ± 1.9	11.2 ± 2.3	10.5 ± 2.2	9.7 ± 2.3	< 0.001
Hematocrit (%)	38.5 ± 5.8	34.6 ± 7.1	32.5 ± 6.7	30.5 ± 7.0	< 0.001
Platelet count (10^9^/L)	201.0 (153.0, 257.0)	198.0 (139.0, 261.0)	190.0 (129.0, 278.2)	184.0 (113.0, 287.0)	0.012
Anion gap (mmol/L)	17.4 ± 5.0	17.2 ± 5.2	17.2 ± 5.3	16.5 ± 5.0	0.005
Serum bicarbonate (mmol/L)	22.9 ± 5.0	22.2 ± 5.5	22.0 ± 5.7	21.1 ± 5.7	< 0.001
Creatinine (mEq/L)	1.2 (0.9, 1.6)	1.3 (0.9, 2.1)	1.5 (1.0, 2.5)	1.5 (1.0, 2.5)	< 0.001
BUN (mg/dl)	23.0 (17.0, 34.0)	30.0 (19.0, 48.0)	34.5 (22.0, 54.0)	35.0 (21.0, 55.0)	< 0.001
Glucose (mg/dL)	140.0 (113.0, 188.0)	139.0 (113.0, 181.8)	136.0 (108.0, 182.2)	125.0 (99.0, 166.0)	< 0.001
Chloride (mmol/L)	100.9 ± 6.1	102.3 ± 7.0	102.0 ± 8.4	103.5 ± 7.8	< 0.001
Sodium (mmol/L)	138.0 ± 5.8	138.2 ± 6.1	137.9 ± 7.5	137.9 ± 6.7	0.879
Potassium (mmol/L)	4.4 ± 1.0	4.5 ± 1.0	4.5 ± 0.9	4.4 ± 0.9	0.151
INR	1.7 ± 1.4	2.0 ± 1.7	2.0 ± 1.8	2.1 ± 1.8	< 0.001
PT (second)	13.9 (12.3, 18.4)	15.3 (13.4, 21.5)	15.8 (13.5, 22.2)	16.7 (14.1, 23.0)	< 0.001
RAR	3.6 ± 0.3	4.5 ± 0.2	5.4 ± 0.3	7.6 ± 2.0	< 0.001
RDW (10^9^/L)	13.9 ± 1.0	14.9 ± 1.5	16.0 ± 1.9	17.8 ± 3.0	< 0.001
Serum albumin (g/dL)	3.9 ± 0.4	3.4 ± 0.3	3.0 ± 0.4	2.4 ± 0.5	< 0.001
*Comorbidities, n*					
Myocardial infarct	191 (25.1)	192 (25.3)	187 (24.6)	163 (21.4)	0.254
Congestive heart failure	366 (48.1)	410 (53.9)	403 (53)	365 (48)	0.027
Peripheral vascular disease	103 (13.5)	108 (14.2)	128 (16.8)	129 (17)	0.14
Cerebrovascular disease	175 (23)	128 (16.8)	109 (14.3)	108 (14.2)	< 0.001
Dementia	54 (7.1)	65 (8.6)	58 (7.6)	43 (5.7)	0.171
Chronic pulmonary disease	197 (25.9)	283 (37.2)	246 (32.4)	210 (27.6)	< 0.001
Rheumatic disease	23 (3)	29 (3.8)	39 (5.1)	31 (4.1)	0.213
Peptic ulcer disease	13 (1.7)	26 (3.4)	37 (4.9)	46 (6)	< 0.001
Liver disease	69 (9.1)	117 (15.4)	118 (15.5)	197 (25.9)	< 0.001
Diabetes	245 (32.2)	252 (33.2)	269 (35.4)	274 (36)	0.346
Renal disease	191 (25.1)	257 (33.8)	284 (37.4)	285 (37.5)	< 0.001
Malignant cancer	62 (8.1)	88 (11.6)	112 (14.7)	179 (23.5)	< 0.001
Metastatic solid tumor	18 (2.4)	31 (4.1)	48 (6.3)	74 (9.7)	< 0.001
Aids	4 (0.5)	1 (0.1)	1 (0.1)	1 (0.1)	0.475
*Organ support therapy, n*					
MV	339 (44.5)	340 (44.7)	348 (45.8)	368 (48.4)	0.419
RRT	28 (3.7)	46 (6.1)	72 (9.5)	94 (12.4)	< 0.001
*Scoring systems*					
OASIS	36.5 ± 8.8	38.2 ± 9.3	39.1 ± 9.8	40.8 ± 9.7	< 0.001
SAPSII	41.5 ± 12.4	45.2 ± 13.5	47.6 ± 14.5	50.9 ± 15.2	< 0.001
SOFA	6.5 ± 3.4	7.5 ± 3.9	8.1 ± 4.3	9.1 ± 4.4	< 0.001
*Outcomes*					
In-hospital mortality, n	139 (18.3)	165 (21.7)	190 (25)	295 (38.8)	< 0.001

*RAR* red blood cell distribution width/albumin ratio,* SBP* systolic blood pressure,* DBP* diastolic blood pressure,* MBP* mean blood pressure,* SPO*_2_ percutaneous oxygen saturation,* WBC* white blood cell,* RDW* red cell distribution width,* INR* international normalized ratio,* PT* plasma prothrombin time,* BUN* blood urea,* MV* mechanical ventilation,* RRT* renal replacement therapy,* OASIS* Oxford acute severity of illness score,* SAPS II* simplified acute physiology score,* SOFA* sequential organ failure assessment

*P*-values were calculated using chi-square test, one-way ANOVA, and Kruskal–Wallis test

### Association between RAR and in-hospital mortality

**Fig. 2:**
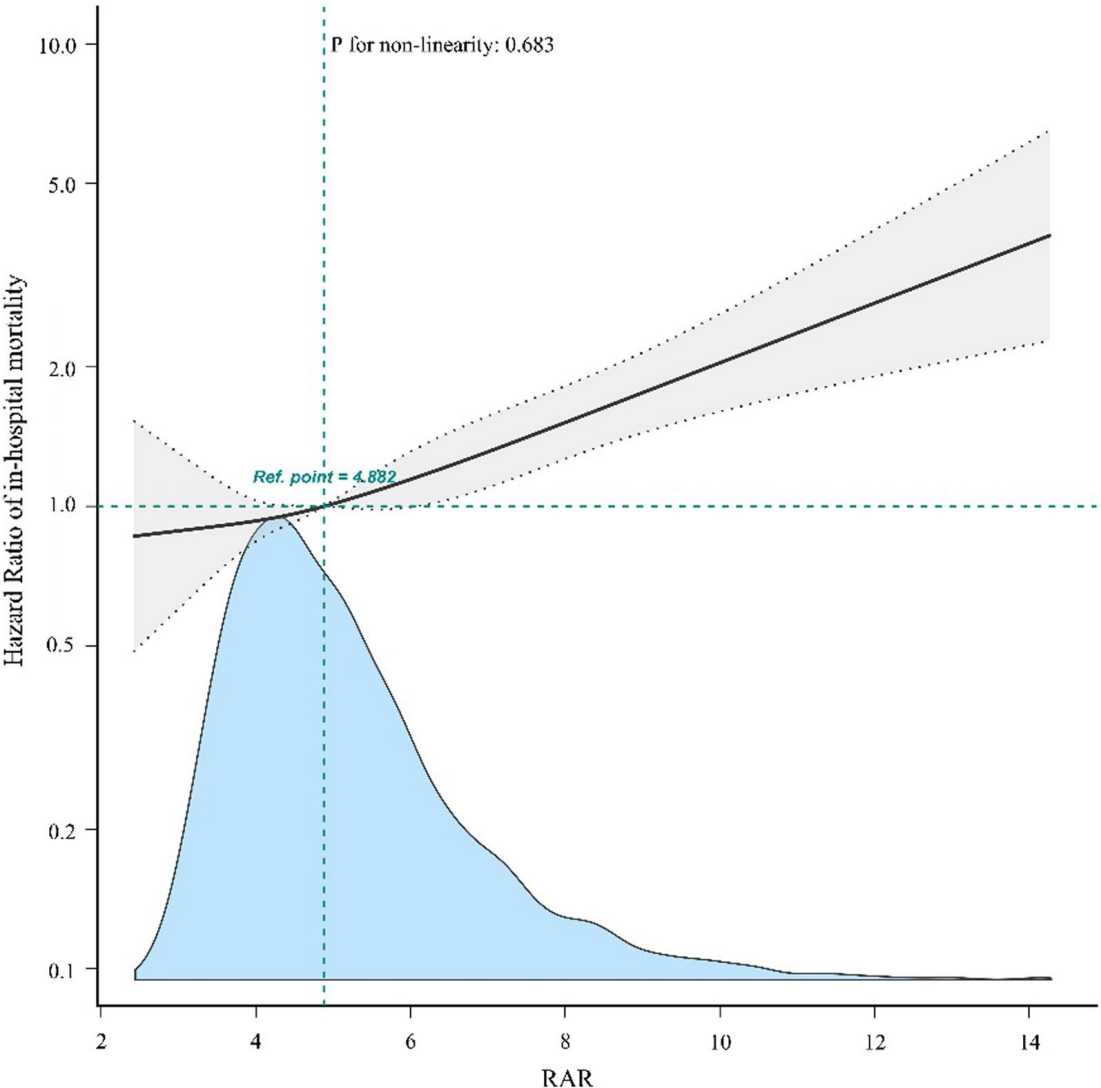
Association between RAR and in-hospital mortality of patients with sepsis and atrial fibrillation. RAR was entered as a continuous variable. Hazard ratios were adjusted for age, sex, MI, CHF, cerebrovascular disease, chronic pulmonary disease, rheumatic disease, diabetes, renal disease, liver disease, MV, RRT, serum hematocrit, MBP, temperature, SPO_2_, potassium, INR, PT, and SOFA score. *RAR* red blood cell distribution width-to-albumin ratio,* MI* myocardial infarction,* CHF* congestive heart failure,* MV* mechanical ventilation, * RRT* renal replacement therapy,* MBP* mean blood pressure, * SPO*_2_ percutaneous oxygen saturation, * INR* international normalized ratio, * PT* plasma prothrombin time, * SOFA* Sequential Organ Failure Assessment. The black and blue lines represent the estimated values and their corresponding 95% confidence intervals, respectively

Age, WBC, heart rate, blood pressure, anion gap, serum bicarbonate, BUN, potassium, INR, PT, RAR, MI, chronic liver disease, metastatic solid tumor, MV, RRT, OASIS, SAPSII, and SOFA scores were all significantly associated with in-hospital mortality in patients with sepsis and AF (Additional file 1: Table S1). A linear relationship was observed between the RAR and in-hospital mortality in patients with sepsis and AF (Fig. 2).

The hazard ratios (HRs) of RAR were consistently significant in all models when the RAR was analyzed as a continuous variable (range 1.03–1.08, *p* < 0.001). When RAR was analyzed as quartiles in model 1, the highest RAR (Q4 vs. Q1) was associated with a higher risk of in-hospital mortality after adjusting for age and sex (adjusted hazard ratio [aHR], 1.76; 95% confidence interval [CI], 1.43–2.15; *p* < 0.001). In model 2, after adjusting for model 1 and MI, CHF, cerebrovascular disease, chronic pulmonary disease, rheumatic disease, diabetes, renal disease, liver disease, MV, and RRT, the HR and 95% CI were 1.65 (1.34–2.04) (*p* < 0.001) for the highest RAR group. Finally, in model 3, after adjusting for model 1, model 2, serum hematocrit, MBP, temperature, SPO_2_, potassium, INR, PT, and SOFA score, the highest RAR was still statistically associated with an increased risk of in-hospital mortality (HR: 1.52, 95% CI: 1.2–1,91, *p* < 0.001). The statistical results were robust among all the models (Table 2).

**Table 2:** Multivariate cox regression of the association between different RAR levels and in-hospital mortality

Outcomes	Crude mode		Model 1		Model 2		Model 3	
	HR (95% CIs)	*P* value	HR (95% CIs)	*P* value	HR (95% CIs)	*P* value	HR (95% CIs)	*P* value
RAR	1.05 (1.03 ~ 1.07)	< 0.001	1.06 (1.04 ~ 1.07)	< 0.001	1.05 (1.03 ~ 1.07)	< 0.001	1.06 (1.03 ~ 1.08)	< 0.001
*Quintiles*								
Q1 (< 4.06)	1(Ref)		1(Ref)		1(Ref)		1(Ref)	
Q2 (4.06–4.89)	1.09 (0.87 ~ 1.36)	0.474	1.09 (0.87 ~ 1.36)	0.478	1.07 (0.85 ~ 1.34)	0.56	1.05 (0.83 ~ 1.33)	0.668
Q3 (4.89-6.0)	1.2 (0.96 ~ 1.49)	0.104	1.24 (1 ~ 1.55)	0.054	1.23 (0.98 ~ 1.53)	0.072	1.15 (0.91 ~ 1.46)	0.233
Q4 (≥ 6.0)	1.65 (1.35 ~ 2.03)	< 0.001	1.76 (1.43 ~ 2.15)	< 0.001	1.65 (1.34 ~ 2.04)	< 0.001	1.52 (1.2 ~ 1,91)	< 0.001
*P* for trend		< 0.001		< 0.001		< 0.001		< 0.001

Cox proportional hazard regression models were used to calculate hazard ratios (HRs) with 95% confidence intervals

Crude model was adjusted for none

Model 1 was adjusted for age and gender

Model 2 was adjusted for model 1+ (MI, CHF, cerebrovascular disease, chronic pulmonary disease, rheumatic disease, diabetes, renal disease, liver disease, MV, and RRT).

Model 3 was adjusted for model 1 + model 2+ (serum hematocrit, MBP, temperature, SPO_2_, potassium, INR, PT, and SOFA).

MI, myocardial infarction; CHF, congestive heart failure; MV, mechanical ventilation; RRT, renal replacement therapy; MBP, mean blood pressure; SPO_2_, percutaneous oxygen saturation; INR, international normalized ratio; PT, prothrombin time; SOFA, Sequential Organ Failure Assessment

**Fig. 3:**
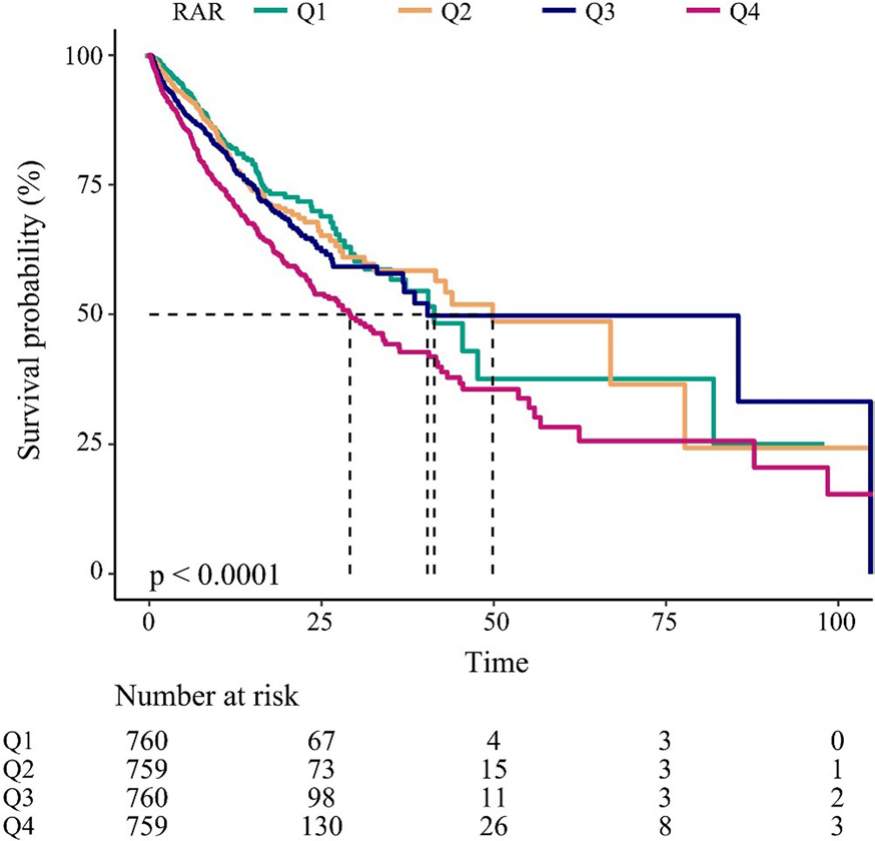
Kaplan–Meier survival analysis of in-hospital survival within different quartiles. Q1, RAR < 4.06; Q2, 4.06 ≤ RAR < 4.89; Q3, 4.89 ≤ RAR < 6.0; Q4, RAR ≥ 6.0; . RAR, red blood cell distribution width-to-albumin ratio

The Kaplan–Meier survival curves comparing patients with different RAR (Fig. 3) showed that patients in the highest RAR quartile (Q4) had the lowest survival among all groups, which declined with declining baseline RAR (*p* < 0.0001).

### Subgroup analyses by adjusted potential effect confounders

**Fig. 4:**
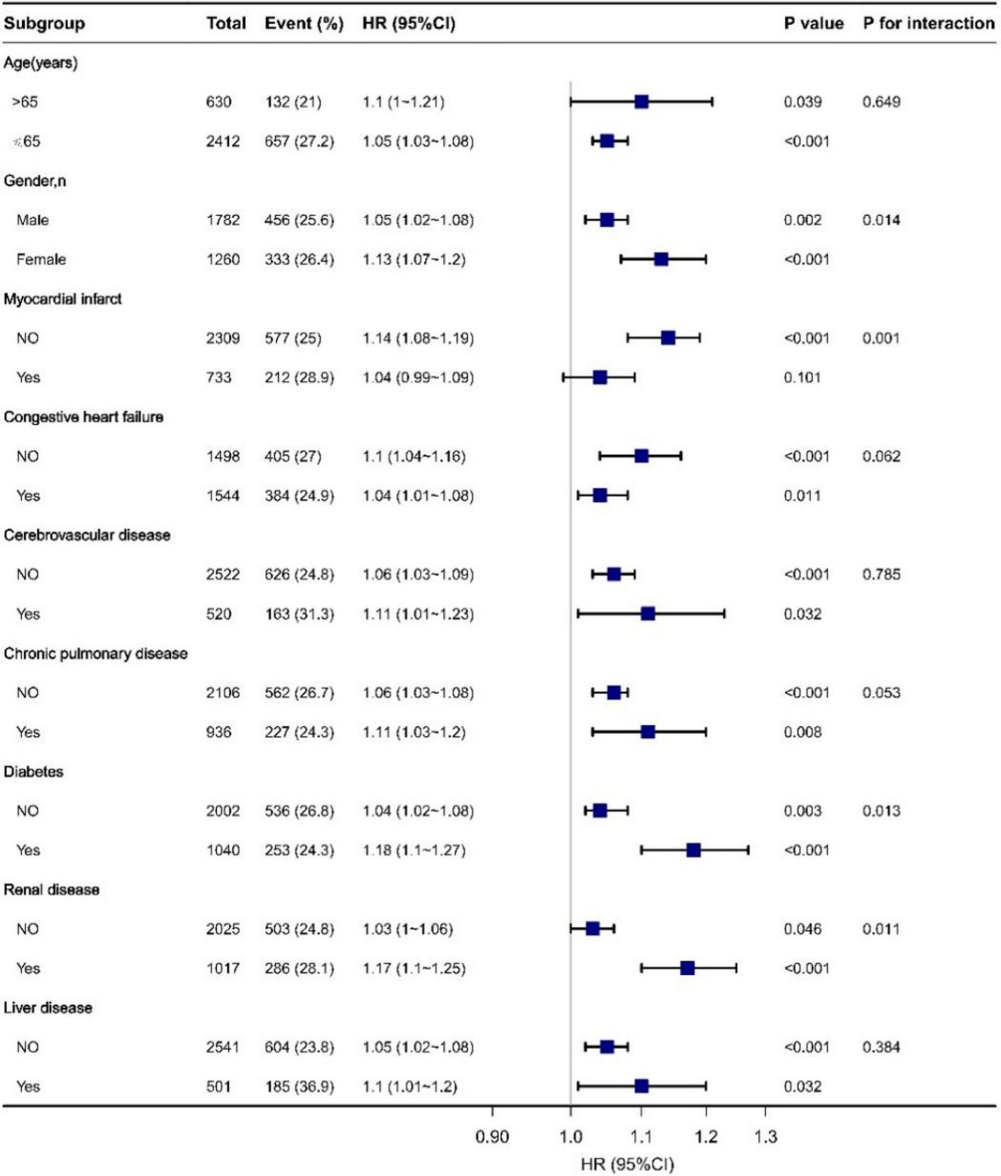
Subgroup analyses of RAR in patients with sepsis and atrial fibrillation. Hazard ratios (HRs) were adjusted for age, sex, MI, CHF, cerebrovascular disease, chronic pulmonary disease, rheumatic disease, diabetes, renal disease, liver disease, MV, RRT, serum hematocrit, MBP, temperature, SPO_2_, potassium, INR, PT, and SOFA score. RAR, red blood cell distribution width-to-albumin ratio; MI, myocardial infarction; CHF, congestive heart failure; MV, mechanical ventilation; RRT, renal replacement therapy; MBP, mean blood pressure; SPO_2_, percutaneous oxygen saturation; INR, international normalized ratio; PT, plasma prothrombin time; SOFA, Sequential Organ Failure Assessment

Stratified analyses were performed to examine whether the association between serum RAR and in-hospital mortality in patients with sepsis and AF was stable among the distinct subgroups (Fig. 4). The data showed significant interactions between RAR and sex, MI, diabetes, and renal failure (all *p* < 0.05). There were no significant associations among those with or without comorbidities (CHF, cerebrovascular disease, chronic pulmonary disease, and liver disease), and similar results were found for age (all *p* > 0.05).

### Sensitivity analysis

In the sensitivity analysis, we excluded 38 patients who had been diagnosed with hepatorenal syndrome before ICU admission. The association between RAR and mortality remained reliable (Additional file 2: Table S2).

## Discussion

To our knowledge, this is the first study to explore the connection between RAR and AF in patients with sepsis. Elevated RAR levels were significantly associated with an increased risk of in-hospital mortality in patients with sepsis and AF.

RDW is known to reflect changes in RBC volume and function. A high RDW is associated with all-cause mortality in cardiovascular and thrombotic diseases, including coronary artery disease, acute and chronic heart failure, and peripheral arterial disease [22]. A study of 69,412 patients with AF showed that dynamic changes in RDW were strongly associated with the risk of all-cause mortality. In patients with elevated RDW, the risk of mortality decreased when RDW declined to normal levels, and in patients with normal RDW, the risk of mortality increased with RDW elevation. Changes in RDW over time were also found to be associated with all-cause mortality in patients with CHF [23]. In addition, a recent study revealed that increased RDW was associated with both in-hospital mortality and short- and long-term mortality in critically ill patients with AF [24]. Erythrocytes deliver oxygen to tissue cells and release mediators for cardiovascular regulation [25]. Thus, alterations in RBCs have a predisposing and exacerbating effect on cardiovascular diseases. Persistently increased RDW is associated with pathophysiological processes involving oxygen deficit and inflammation [26]. The hormone erythropoietin, secreted during hypoxia, promotes the release of enlarged RBCs, leading to an abnormal increase in RDW in cardiovascular diseases [27]. During inflammation, cytokines, such as tumor necrosis factor and interleukins, can hinder RBC production, promote RBC apoptosis, induce abnormal RBC membranes, and reduce iron utilization [5, 28]. The RBC is damaged, and the RBC maturation cycle is prolonged, leading to an increase in the heterogeneity of RBCs in the peripheral blood, which manifests as an increase in RDW.

Serum albumin is commonly examined in hospitalized patients. Serum albumin plays a role in the acute response to inflammation [29] and it has been suggested as a reliable predictor of outcomes in critically ill patients with infections [30, 31]. Previous studies have shown that low serum albumin level at sepsis presentation is a strong predictor of septic shock [32]. Further studies have suggested that mortality in hospitalized patients is associated with hypoalbuminemia, which has been shown to increase mortality [33]. Finfer et al. found that patients with severe sepsis receiving albumin had a lower risk of death than those receiving normal saline, though this difference was not statistically significant [34]. Normal concentrations of serum albumin may scavenge peroxyl radicals [35], inhibit platelet activation and aggregation [36], and improve blood viscosity [37]. Serum albumin plays anti-inflammatory and antithrombotic roles in this process. Once the serum albumin decreases, the disease process worsens.

However, RAR, as a combined inflammation-related index, is stable and easily accessible. As such, RAR may be a better tool than other single-identified markers (RDW and albumin) for assessing inflammatory responses. Previous research has indicated that RAR is associated with 60-day mortality in patients with ARDS [16]. A higher RAR was significantly associated with increased 28-day mortality (odds ratio [OR] 1.338, 95% CI 1.094–1.637, *p* = 0.005), which is similar to the lactate/albumin ratio in critically-ill patients with pneumonia receiving invasive MV [38]. Lu et al. recently observed that an increased RAR ratio was independently associated with increased all-cause mortality in patients with cancer [18]. A study showed that RAR is a potential diagnostic and prognostic marker in patients with cardiovascular diseases. High levels of RAR are associated with increased short- and long-term mortality in patients with heart failure [13].

In our study, we recruited 3042 individuals. After controlling for a predefined set of confounding variables, Cox regression and Kaplan**–**Meier survival curves both showed that higher RAR is related to increased mortality. In the sensitivity analysis, no difference was found after excluding the 38 patients with hepatorenal syndrome before ICU admission. Moreover, the subgroup analysis results indicated that more attention should be paid to high-risk patients, including women, patients without MI, patients with diabetes, and patients with renal disease. Based on the existing putative mechanisms, we may understand the association between RAR and sepsis and AF. First, sepsis and AF are related to inflammatory diseases, leading to increased RDW elevation and decreased albumin levels. Second, aberrant RDW and albumin levels were associated with accelerated disease progression. Abnormalities in hematological indicators were predisposing factors for disease. In contrast, RAR is a useful prognostic indicator of disease progression. Moreover, RAR is inexpensive, quickly available from laboratories, and can be widely used, especially in less-developed areas.

This study had several limitations. First, causality could not be determined due to the observational study design. Second, our study was a single-center retrospective study, and the findings should be further confirmed by multicenter prospective studies. Third, because the pathophysiology and other clinical characteristics are not readily available in MIMIC IV, we were unable to distinguish new-onset AF during ICU admission from chronic AF before ICU stay.

## Conclusion

We provide the first evidence that a high RAR level is associated with increased in-hospital mortality in patients with sepsis and AF. Elevated RAR was significantly associated with elevated risk in these patients.

## Supplementary Information

**Additional file 1**: **Table S1**. Factors related to in-hospital mortality in red blood cell distribution width-to-albumin ratio by univariate analysis.

**Additional file 2**: **Table S2**. Multivariate Cox regression analyses of the association between different red blood cell distribution width-to-albumin ratio levels and in-hospital mortality after excluding patients with hepatorenal syndrome before intensive care unit admission.

## Data Availability

Data used to support the findings of this study are available from the corresponding author upon request.

## Abbreviations

RAR: Red blood cell distribution width-to-albumin ratio
AF: Atrial fibrillation
RDW: Red cell distribution width
ARDS: Acute respiratory distress syndrome
MIMIC: Medical information mart for intensive care
ICU: Intensive care unit
SOFA: Sequential organ failure assessment
SBP: Systolic blood pressure
DBP: Diastolic blood pressure
SPO_2_: Percutaneous oxygen saturation
WBC: White blood cell
RDW: Red cell distribution width
INR: International normalized ratio
PT: Plasma prothrombin time
BUN: Blood urea
MV: Mechanical ventilation
RRT: Renal replacement therapy
OASIS: Oxford acute severity of Illness score
SAPS II: Simplified acute physiology score
CHF: Congestive heart failure
MI: Myocardial infarct
SD: Standard deviation
IQR: Interquartile range
HR: Hazard ratio
CI: Confidence interval
RBC: Red blood cell
