# Supplementary material for: Relationship between red blood cell distribution width-to-albumin ratio and outcome of septic patients with atrial fibrillation: a retrospective cohort study
Source: BMC Cardiovasc Disord. 2022 Dec 9;22:538. doi: 10.1186/s12872-022-02975-1 (PMC9733276; doi:10.1186/s12872-022-02975-1)
Supplement: Supplementary file 1 — Additional file 1: Table S1. Factors related to in-hospital mortality in red blood cell distribution width-to-albumin ratio by univariate analysis. [file 12872_2022_2975_MOESM1_ESM.docx]

**Additional file 1:**

**Table S1.** Factors related to in-hospital mortality in red blood cell distribution width-to-albumin ratio by univariate analysis.

| Variables | Statistics | HR (95%CI) | *P* value |
| --- | --- | --- | --- |
| Age (years) | 74.9 ± 12.3 | 1.03 (1.02,1.03) | < 0.001 |
| Sex, n |  |  |  |
| Male | 1782 (58.6) | Ref |  |
| Female | 1260 (41.4) | 0.99 (0.86,1.13) | 0.836 |
| Heart rate (beats/min) | 93.1 ± 22.9 | 1.00 (1.00,1.01) | 0.028 |
| SBP (mmHg) | 120.9 ± 25.0 | 1.00 (1.00,1.00) | 0.001 |
| DBP (mmHg) | 67.7 ± 19.1 | 1.00 (1.00,1.00) | 0.008 |
| MBP (mmHg) | 82.0 ± 20.0 | 1.00 (1.00,1.00) | 0.001 |
| Temperature (℃) | 36.7 ± 0.9 | 0.84 (0.79,0.89) | < 0.001 |
| SPO_2_ (%) | 96.0 ± 5.0 | 0.97 (0.96,0.98) | < 0.001 |
| WBC count (10^9^/L) | 12.1 (8.1, 17.3) | 1.00 (1.00,1.01) | 0.095 |
| Hemoglobin (g/dl) | 11.0 ± 2.4 | 0.99 (1.00,1.02) | 0.526 |
| Hematocrit (%) | 34.1 ± 7.3 | 1.00 (1.00,1.01) | 0.555 |
| Platelet count (10^9^/L) | 195.0 (134.0, 269.0) | 1.00 (1.00,1.00) | 0.88 |
| Anion gap (mmol/L) | 17.1 ± 5.1 | 1.05 (1.03,1.06) | < 0.001 |
| Serum bicarbonate (mmol/L) | 22.0 ± 5.5 | 0.96 (0.95,0.98) | < 0.001 |
| Creatinine (mEq/L) | 1.4 (1.0, 2.2) | 1.04 (1.00,1.07) | 0.053 |
| BUN (mg/dl) | 29.5 (19.0, 49.0) | 1.01 (1.00,1.01) | < 0.001 |
| Glucose (mg/dL) | 135.0 (108.0, 179.0) | 1.00 (1.00,1.00) | 0.19 |
| Chloride (mmol/L) | 102.2 ± 7.4 | 1.00 (1.00,1.01) | 0.368 |
| Sodiumn (mmol/L) | 138.0 ± 6.5 | 1.00 (1.00,1.01) | 0.897 |
| Potassium (mmol/L) | 4.4 ± 1.0 | 1.18 (1.1,1.26) | < 0.001 |
| INR | 2.0 ± 1.7 | 1.07 (1.04,1.1) | < 0.001 |
| PT (second) | 15.4 (13.2, 21.5) | 1.01 (1.00,1.01) | < 0.001 |
| RAR | 5.3 ± 1.8 | 1.05 (1.03,1.07) | < 0.001 |
| RDW (10^9^/L) | 15.7 ± 2.5 | 1.06 (1.03,1.09) | < 0.001 |
| Serum albumin (g/dL) | 3.2 ± 0.7 | 0.78 (0.7,0.86) | < 0.001 |
| Myocardial infarct |  |  |  |
| NO | 2309 (75.9) | Ref |  |
| Yes | 733 (24.1) | 1.18 (1.01,1.38) | 0.04 |
| Congestive heart failure |  |  |  |
| NO | 1498 (49.2) | Ref |  |
| Yes | 1544 (50.8) | 0.96 (0.83,1.1) | 0.536 |
| Peripheral vascular disease |  |  |  |
| NO | 2574 (84.6) | Ref |  |
| Yes | 468 (15.4) | 1.01 (0.84,1.22) | 0.9 |
| Cerebrovascular disease |  |  |  |
| NO | 2522 (82.9) | Ref |  |
| Yes | 520 (17.1) | 1.13 (0.95,1.34) | 0.177 |
| Dementia |  |  |  |
| NO | 2822 (92.8) | Ref |  |
| Yes | 220 (7.2) | 1.25 (0.95,1.63) | 0.106 |
| Chronic pulmonary disease |  |  |  |
| NO | 2106 (69.2) | Ref |  |
| Yes | 936 (30.8) | 0.92 (0.79,1.08) | 0.308 |
| Rheumatic disease |  |  |  |
| NO | 2920 (96.0) | Ref |  |
| Yes | 122 (4.0) | 1.3 (0.92,1.82) | 0.133 |
| Peptic ulcer disease |  |  |  |
| NO | 2920 (96.0) | Ref |  |
| Yes | 122 (4.0) | 0.84 (0.59,1.17) | 0.3 |
| Liver disease |  |  |  |
| NO | 2541 (83.5) | Ref |  |
| Yes | 501 (16.5) | 1.21 (1.03,1.43) | 0.023 |
| Diabetes |  |  |  |
| NO | 2002 (65.8) | Ref |  |
| Yes | 1040 (34.2) | 0.88 (0.76,1.02) | 0.094 |
| Renal disease |  |  |  |
| NO | 2025 (66.6) | Ref |  |
| Yes | 1017 (33.4) | 1.11 (0.96,1.29) | 0.149 |
| Malignant cancer |  |  |  |
| NO | 2601 (85.5) | Ref |  |
| Yes | 441 (14.5) | 1.05 (0.87,1.27) | 0.606 |
| Metastatic solid tumor |  |  |  |
| NO | 2871 (94.4) | Ref |  |
| Yes | 171 (5.6) | 1.67 (1.31,2.14) | < 0.001 |
| Aids |  |  |  |
| NO | 3035 (99.8) | Ref |  |
| Yes | 7 (0.2) | 0.62 (0.09,4.41) | 0.634 |
| MV |  |  |  |
| NO | 1647 (54.1) | Ref |  |
| Yes | 1395 (45.9) | 1.57 (1.36,1.81) | < 0.001 |
| RRT |  |  |  |
| NO | 2802 (92.1) | Ref |  |
| Yes | 240 (7.9) | 1.32 (1.06,1.63) | 0.011 |
| OASIS | 38.7 ± 9.5 | 1.05 (1.04,1.06) | < 0.001 |
| SAPS Ⅱ | 46.3 ± 14.3 | 1.03 (1.03,1.04) | < 0.001 |
| SOFA | 7.8 ± 4.1 | 1.1 (1.08,1.11) | < 0.001 |

RAR, red blood cell distribution width/albumin ratio; SBP, systolic blood pressure; DBP, diastolic blood pressure; MBP, mean blood pressure; SPO_2_, percutaneous oxygen saturation; WBC, white blood cell; RDW, red cell distribution width; INR, international normalized ratio; PT, plasma prothrombin time; BUN, blood urea; MV, mechanical ventilation; RRT, renal replacement therapy; OASIS, Oxford acute severity of illness score; SAPS II, simplified acute physiology score; SOFA, sequential organ failure assessment.
