# Supplementary material for: Relationship between red blood cell distribution width-to-albumin ratio and outcome of septic patients with atrial fibrillation: a retrospective cohort study
Source: BMC Cardiovasc Disord. 2022 Dec 9;22:538. doi: 10.1186/s12872-022-02975-1 (PMC9733276; doi:10.1186/s12872-022-02975-1)
Supplement: Supplementary file 2 — Additional file 2: Table S2. Multivariate Cox regression analyses of the association between different red blood cell distribution width-to-albumin ratio levels and in-hospital mortality after excluding patients with hepatorenal syndrome before intensive care unit admission. [file 12872_2022_2975_MOESM2_ESM.docx]

**Additional file 2:**

**Table S2.** Multivariate Cox regression analyses of the association between different red blood cell distribution width-to-albumin ratio levels and in-hospital mortality after excluding patients with hepatorenal syndrome before intensive care unit admission.

| Outcomes | Crude mode | | Model 1 | | Model 2 | | Model 3 | |
| --- | --- | --- | --- | --- | --- | --- | --- | --- |
|  | HR (95% CIs) | *P* value | HR (95% CIs) | *P* value | HR (95% CIs) | *P* value | HR (95% CIs) | *P* value |
| RAR | 1.05 (1.03~1.07) | <0.001 | 1.05 (1.03~1.07) | <0.001 | 1.05 (1.02~1.07) | <0.001 | 1.05 (1.03~1.08) | <0.001 |
| Quintiles |  |  |  |  |  |  |  |  |
| Q1 (<4.06) | 1(Ref) |  | 1(Ref) |  | 1(Ref） |  | 1(Ref） |  |
| Q2 (4.06-4.89) | 1.12 (0.89~1.40) | 0.336 | 1.10 (0.88~1.38) | 0.400 | 1.09 (0.86~1.37) | 0.478 | 1.06 (0.84~1.35) | 0.614 |
| Q 3 (4.89-6.0) | 1.19 (0.95~1.49) | 0.120 | 1.22 (0.98~1.52) | 0.079 | 1.21 (0.96~1.51) | 0.099 | 1.12 (0.88~1.42) | 0.355 |
| Q 4 (≥6.0) | 1.62 (1.32~2.20) | <0.001 | 1.72 (1.40~2.11) | <0.001 | 1.61 (1.30~1.99) | <0.001 | 1.46 (1.16~1.85) | 0.001 |
| *P* for trend |  | <0.001 |  | <0.001 |  | <0.001 |  | 0.001 |
